# Access Control Mechanism for IoT Environments Based on Modelling Communication Procedures as Resources

**DOI:** 10.3390/s18030917

**Published:** 2018-03-20

**Authors:** Luis Cruz-Piris, Diego Rivera, Ivan Marsa-Maestre, Enrique de la Hoz, Juan R. Velasco

**Affiliations:** Departamento de Automática, Escuela Politécnica Superior, Universidad de Alcalá, 28805 Alcalá de Henares, Madrid, Spain; diego.rivera@uah.es (D.R.); ivan.marsa@uah.es (I.M.-M.); enrique.delahoz@uah.es (E.d.l.H.); juanramon.velasco@uah.es (J.R.V.)

**Keywords:** access control, Internet of Things, security, MQTT, OAuth

## Abstract

Internet growth has generated new types of services where the use of sensors and actuators is especially remarkable. These services compose what is known as the Internet of Things (IoT). One of the biggest current challenges is obtaining a safe and easy access control scheme for the data managed in these services. We propose integrating IoT devices in an access control system designed for Web-based services by modelling certain IoT communication elements as resources. This would allow us to obtain a unified access control scheme between heterogeneous devices (IoT devices, Internet-based services, etc.). To achieve this, we have analysed the most relevant communication protocols for these kinds of environments and then we have proposed a methodology which allows the modelling of communication actions as resources. Then, we can protect these resources using access control mechanisms. The validation of our proposal has been carried out by selecting a communication protocol based on message exchange, specifically Message Queuing Telemetry Transport (MQTT). As an access control scheme, we have selected User-Managed Access (UMA), an existing Open Authorization (OAuth) 2.0 profile originally developed for the protection of Internet services. We have performed tests focused on validating the proposed solution in terms of the correctness of the access control system. Finally, we have evaluated the energy consumption overhead when using our proposal.

## 1. Introduction

Internet-based services have evolved fast in the last decade, encouraging the creation of new technologies and the development of existing ones. Furthermore, massive use of the Internet has resulted in an increased number of data generators and consumers, making it especially important to meet the challenges of protection and access control for these data [1].

Parallel to the growth of Internet-based services, the interconnection of devices, sensors, and other objects has resulted in the Internet of Things (IoT) paradigm [2]. Machine to Machine (M2M) protocols have arisen in order to meet the needs of interconnecting local environments, and have been used as the foundation of this new model.

Initially, IoT protocols were used to collect data generated by autonomous devices (e.g., for storage), or to request simple actions to be performed. Therefore, the main design goal in this context has been keeping communications as lightweight as possible, given the constraints of the involved devices in terms of autonomy, memory, computational capabilities, etc., and of the underlying networks [3]. Because of this, these protocols prioritized characteristics such as simplicity or lightness versus other characteristics such as security. This has produced a slower evolution with respect to these issues compared to the Internet protocols.

In this work, we propose modelling the main constituent elements of Internet communication protocols as resources that can be protected by using the access control schemes created for Internet-based services. To achieve this, after studying the main communications protocols, and the most important access control schemes which can be used in IoT environments, we have selected a schema based on the Open Authorization (OAuth) 2.0 profile called User-Managed Access (UMA) [4], designed mainly for Web-based services, which offers a great deal of granularity in access control. The OAuth 2.0 protocol [5] is a de facto standard to solve this situation, while offering a high level of granularity and ease of use. The main component in OAuth is the authorization server, which performs the access control tasks. Regarding communication protocols, we have chosen Message Queuing Telemetry Transport (MQTT) [6] because it is one of the most used communication protocols in IoT and uses a publish/subscribe pattern, which is different from the usual request/response scheme followed by Internet services. An initial proposal of this work was presented in the 5th International Symposium on Sensor Science (I3S 2017) [7].

One of the main challenges of an authorization system for IoT is dealing with the complexity of this kind of environment. The main advantages of the unified authorization system that we present in this work are the easier management of resource protection and the ability to apply the same access control rules defined for Web Services to IoT elements. This paper contributes to this goal in the following ways:

- We analyse the different protocol types used widely in these kinds of environments, and then the access control schemes proposed for them (Section 2).
- We propose to model the main elements of an IoT communications system such as MQTT as resources that can be protected along with other Internet-based services using a unified authorization system (Section 3).
- We propose the integration of an authorization layer, based on an OAuth 2.0 profile, into the chosen IoT communications protocol. This layer allows us to protect both Internet-based services and sensors/actuators through IoT devices. We describe the steps of each of the phases of the authorization system (Section 3.1 and Section 3.2).
- We define a series of experiments where we describe the entities of our proposal, the test set-up, and the effects, in time and electric consumption overhead of applying our proposal to the protocol (Section 4).

After analysing the results obtained in Section 4.2, we conclude that our proposal is not only feasible but also shows a good performance in the evaluated fields. Finally, we discuss the obtained results, summarize our contributions, and show some future research lines (Section 5).

## 2. Communication Protocols and Access Control Systems in IoT Environments

Due to the growth of the IoT-based environments and their heterogeneity, many proposals for the communication of devices have been proposed, with different degrees of standardization. Communication protocols can be classified using different criteria. The authors of [8] divide the protocols according to a protocol stack like the one used in traditional networks.

Using this classification, it is possible to talk about infrastructure protocols, service discovery protocols, and application protocols. The network infrastructure can also be divided into a physical, link, and network layer. Protocols such as Institute of Electrical and Electronics Engineers (IEEE) 802.15.4 [9] (physical and link layer) or Internet Protocol version 6 (IPv6) over Low power Wireless Personal Area Networks (6LoWPAN) [10] (network layer) can be placed in the infrastructure of IoT. Also, the IPv6 Routing Protocol for Low-Power and Lossy Networks (RPL) [11], a routing protocol based on IPv6 and enforced by the Internet Engineering Task Force (IETF), is the main routing protocol for sensor and IoT networks. Service discovery protocols, such as the multicast Domain Name System (mDNS) or Domain Name System Service Discovery (DNS-SD) [12] are used to discover resources and services offered by IoT devices.

Finally, application protocols are used for the exchange of data between applications on top of the IoT infrastructure. This is the category of protocols which is important for our proposal, as it is in these protocols that the users define the information exchange and therefore, here the data access protection must take place.

These protocols can be classified according how they organize the communication between devices. Many of the protocols are based on a publishing/subscription mechanism, where some devices subscribe to a communication channel, and other devices publish data in it, which are then transmitted to the subscribers. This mechanism is well suited for IoT environments, where usually devices need to be able to provide data without being requested like in traditional request/response Internet protocols. The MQTT protocol, the Advanced Message Queuing Protocol (AMQP) and the Data Distribution Service (DDS) are the main protocols of this kind:

- The MQTT protocol was designed by IBM and aimed to offer lightweight communications for M2M environments. It is based on a broker which manages the subscriptions, and is widely used in IoT environments, due to its low energy consumption and reliability [6].
- The AMQP is an alternative to the MQTT protocol which also offers a publish/subscribe scheme with optional high reliability. It is based on two main components: exchanges (used to route messages) and message queues. It is also based on the use of a broker to manage the queues [13].
- The DDS is another publish/subscribe alternative developed by the Object Management Group (OMG). The main difference between the DDS and MQTT or the AMQP is that the DDS does not require a broker for the management of communications, providing a multicasting architecture with no less reliability and Quality of Service (QoS) than their alternatives [14].

On the other hand, the existence of message exchange protocols does not imply that only this kind of protocol is used in IoT environments. In fact, the ubiquity of the Hypertext Transfer Protocol (HTTP) in Internet services has led it being used also in IoT scenarios. Indeed, many IoT services are defined as RESTful services [15]. Representational State Transfer (REST) is not exactly a protocol, but an architectural proposal for the use of the HTTP methods to provide a resource-oriented system to operate in Internet-based services [16].

There are also proposed protocols which can be said to hold an intermediate position between the message exchange protocols and those based on requests and responses. This is the case of the Constrained Application Protocol (CoAP) and the Extensible Messaging and Presence Protocol (XMPP).

- The CoAP is being developed by the IETF Constrained RESTful Environments (CoRE) group, and defines a message transfer protocol based on REST (and therefore, on HTTP) [17]. One of its main advantages is the possibility of easy interoperation between CoAP and HTTP services. It allows communications using both a publish/subscribe model and a request/response model.
- The XMPP is an IETF standard designed for instant messaging, which can also be used in many IoT scenarios [18]. As with the CoAP, it can be used both with a message exchange or a request/response approach.

Security and privacy are crucial challenges in communication systems, and there is a widespread interest in research on these topics for any communication protocol used nowadays. A very important part of any security system is the access control to the information exchanged, and IoT environments are not an exception to this. The work in [19] provides a very extensive review of access control for IoT proposals and challenges. Specifically, it provides a taxonomy of access control models, protocols, and frameworks used in this kind of scenario.

Regarding access control models, they can be classified according to the element on which they base the access control. For instance, it is possible to talk about Role-Based Access Control (RBAC) models, Attribute-Based Access Control (ABAC) models, Usage Control (UCON) models, Capability-Based Access Control (CapBAC) models, Organizational-Based Access Control (OrBAC) models, and others which do not fit in these categories.

RBAC models [20] are based on the role of users in the system, and the permissions given to them. Although there are some approaches to their use in IoT [21,22], they are models suitable for scenarios where there is an easy identification of permitted tasks for each user or service. ABAC models [23], on the other hand, are based on policies that match certain attributes from the subject. They have been used in IoT in works like [24,25]. UCON models [26] are an evolution of RBAC and ABAC models, offering a more flexible way of handling authorization. CapBAC models [27,28] are based on the capability concept (a ticket or token held by some entity which grants certain permissions). Usually, it relies on an Access Control Matrix (ACM), which can be seen an evolution of traditional Access Control Lists (ACLs). Finally, OrBAC models [29] have been created to offer an extension to RBAC, where they introduce new abstraction levels and concepts to make the model more flexible.

There are also many research and development works that are based on well-known access control schemes, and that propose extending them to be used in IoT environments. The most used access control schemes of this type are the Extensible Access Control Markup Language (XACML) [30], OAuth [31], and more recently, UMA [4].

The XACML is a language based on the eXtensible Markup Language (XML) and is designed specifically to provide a standardized description of access control policies. It includes a scheme on how to use it. It has been used in many IoT-related works such as [32,33].

OAuth is a framework designed to provide an access control scheme to Web Services and applications. It is probably the most used framework in this kind of environment (counting both versions 1.0 and 2.0 [5]), and consequently, a lot of efforts have been made to provide OAuth-based solutions for IoT (for instance, works like [34]). In [35] an implementation can be found for the CoAP, and there is another one for the MQTT protocol in [36]. There are however some issues when applying the OAuth scheme from the Web to the IoT world, and some adaptation is required for it to work with IoT restrictions [37].

UMA is essentially a specification based on OAuth which aims to extend the basic use of the authorization scheme to wider and more heterogeneous scenarios. It has not been defined specifically for IoT, but its flexibility makes it a very interesting approach to be used in those scenarios [38], although it requires an adaptation to the IoT protocols and their characteristics.

Other works follow different approaches. For instance, in [39] the authors propose an authentication service for the CoAP in low-power environments, and in [40] they use the Security Assertion Markup Language (SAML) to provide secure communications over the XMPP. New technological tendencies like blockchain have also been applied to offer distributed access control schemes [41].

UMA is a promising mechanism for access control, and it extends OAuth, one of the most important access control schemes in Internet nowadays, while enabling access control in more diverse scenarios. We have selected it as the base scheme which will be used to protect the IoT communications, after being modelled as resources.

Regarding the IoT application protocol to protect, our proposal is focused on integrating the message exchange protocols with the Internet service ecosystem. Taking this into account, we have chosen one of the most used message exchange protocols from those listed above, MQTT. This does not mean that our proposal could not be adapted for other protocols (i.e., the AMQP or XMPP), but the extensive use of the MQTT protocol in IoT scenarios makes it a suitable choice for our experiments.

In Section 2.1 and Section 2.2, we describe both MQTT and UMA and their components in more detail, to be able to use them to describe our proposal.

### 2.1. MQTT Protocol

The MQTT protocol was designed to exchange telemetry information following a publisher/subscriber architecture in networks composed of constrained devices. This kind of device is usually not able to implement complex application-level protocols like the HTTP. The MQTT protocol was also devised to minimize bandwidth and energy usage (as shown in Section 4.2.1) and to be as simple and easy to integrate as possible. The protocol is usually employed with a central server (named the Broker) that coordinates the publisher/subscriber scheme based on the concept of topics. MQTT topics are labels attached to application messages that are used by the server to route the message to the appropriate subscribers. Despite the benefits described above, the MQTT protocol was not originally designed with security in mind. The core protocol offers very simple security features, like user/password credentials for authentication, and does not specify any authorization model. In the most recent versions of the standard, the committee has included a section with security considerations and recommendations on how to implement security for MQTT. These recommendations are described with respect to the usage of Virtual Private Network (VPN) and Transportation Layer Security (TLS) for the Network and Transport layers, respectively. These protocols are not always a suitable option for resource-constrained devices. The MQTT protocol has recently become an international standard [42].

**Figure 1:**
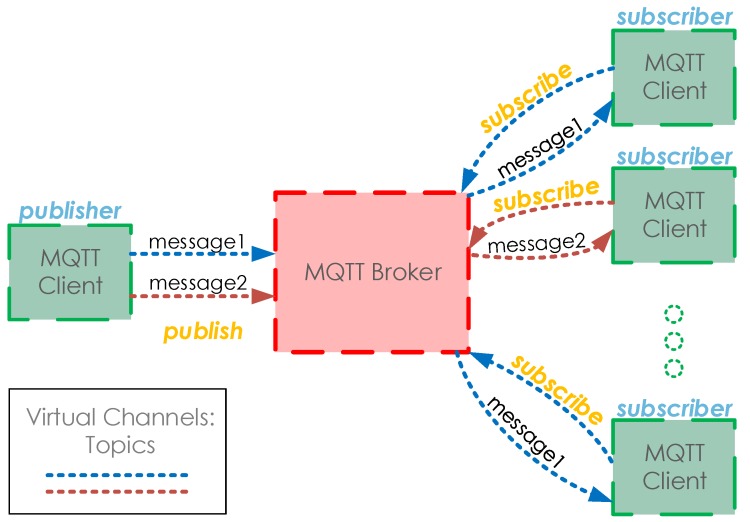
Main entities of the Message Queuing Telemetry Transport (MQTT) protocol.

The main entities in any MQTT system are shown in Figure 1 and can be described as follows:

- Brokers: Central servers in charge of routing and managing the MQTT messages.
- Publishers: Entities that issue messages to subscribing entities through the Broker.
- Subscribers: Entities that receive messages through the Broker. They are subscribed to a topic in the Broker, and receive the messages related to it. An entity can be both a publisher and subscriber.
- Topics: Identifiers of the subject of a series of messages. They can be seen as the channel used by the Publisher and Subscriber to exchange messages. They follow a hierarchical structure, where there are various levels of topics separated by a slash (e.g., /myhouse/kitchen/temperature).

The MQTT protocol does not specify any authorization or access control scheme, and in fact, does not provide a specific authorization header in its specification, but the CONNECT message (issued to establish a new connection with the Broker) contains an optional authentication header, composed of a username and password fields, which are sent in clear text unless some encryption system is enforced over the protocol (usually Secure Sockets Layer (SSL)). Although is not part of the standard specification, it is possible to use this optional header to carry access control information, such as tokens or tickets, instead of the original username/password pair (for instance, it is possible to issue an OAuth access token in the username field, and leave the password field blank). We will take advantage of this possibility to protect the MQTT topics using UMA tokens.

### 2.2. User-Managed Access (UMA)

Although OAuth 2.0 is a widely used protocol in many different services, there are scenarios which cannot be covered by the standard. In fact, the main scenario in OAuth implies an application owner giving access permissions to another application he/she owns. However, there are scenarios where this is not the case, for instance, a user might want to give access permissions to a third party which could use the resources he/she owns (defining resource as any personal data, content, services, etc.), which is not directly possible using OAuth. UMA on the other hand, has been designed specifically to provide a solution for these scenarios. It is being developed by a working group of the Kantara Initiative [43], a non-profit organization composed by different companies and communities. The UMA specification draft has been contributed by the Internet Engineering Task Force (IETF) [44].

In the specification, the UMA schema is defined as composed of a series of entities:

- Resource Owner (RO): The entity in charge of granting or denying access to a protected resource. Usually this role is represented by an end-user.
- Requesting Party (RP): The entity which requests access to the protected resource. As is the case of the RO, is usually represented by an end-user.
- Client: An application used by RPs to make requests in their name to access protected resources owned by any RO.
- Resource Set (RS): A group composed of one or more resources managed together by a Resource Server [45], that is, using the same access policies (which are defined in the Authorization Server) for each resource in the set.
- Resource Server: The entity which implements the management of RS protection by their ROs.
- Authorization Server (AS): The entity in charge of issuing authorization and permission tokens to clients, and which enforces the RS protection at the resource servers. It is composed of two OAuth-protected Application Programming Interfaces (APIs) (Authorization API, and Protection API) and an RS management interface used by ROs.

The whole UMA protection and resource access is based on three tokens, which can be seen in Table 1.

**Figure 2:**
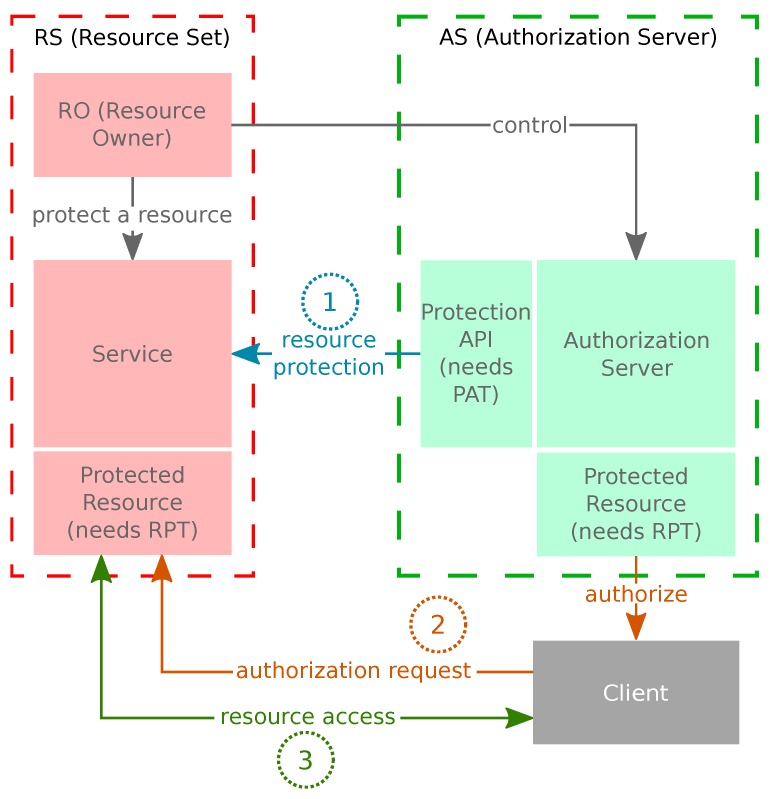
The main phases and entities of User-Managed Access (UMA). PAT: Protection API Token; RPT: Requesting Party Token.

These entities from the UMA schema and its main phases are shown in Figure 2.

The three main phases of UMA are:

1. Resource Protection: The RO of an RS registers it into the AS though the Protection API (using the Protection API Token, PAT). Additionally, the RO configures the access policies for the RS scopes in the RS management interface.
2. Authorization Request: The RP, through a client, requests access to a resource through the Resource Server. It must obtain the authorization data and a Requesting Party Token (RPT) through the Authorization API of the AS (using the Authorization API Token, AAT).
3. Resource Access: Using the RPT and the authorization data associated with it, the RP is able to access the resource through its client.

## 3. Proposed Solution: Modelling Components of a Communication Protocol as Resources

The central component of MQTT architecture is the Broker, which is entitled to receive, process, and re-transmit all MQTT messages (managing publish and subscribe actions) following the protocol specification.

We propose modelling the MQTT Broker as a UMA Resource Server (Resource Server Broker). This way, full compatibility is maintained with the current MQTT protocol specifications, while adding the authorization capabilities offered by UMA and not found in MQTT. MQTT is defined around topics. To provide authorization capabilities over topics, we propose modelling MQTT topics as UMA resources. According to UMA, every resource must have an owner. Therefore, topics will have an owner, which will be responsible of administering the access control policies associated with it.

**Figure 3:**
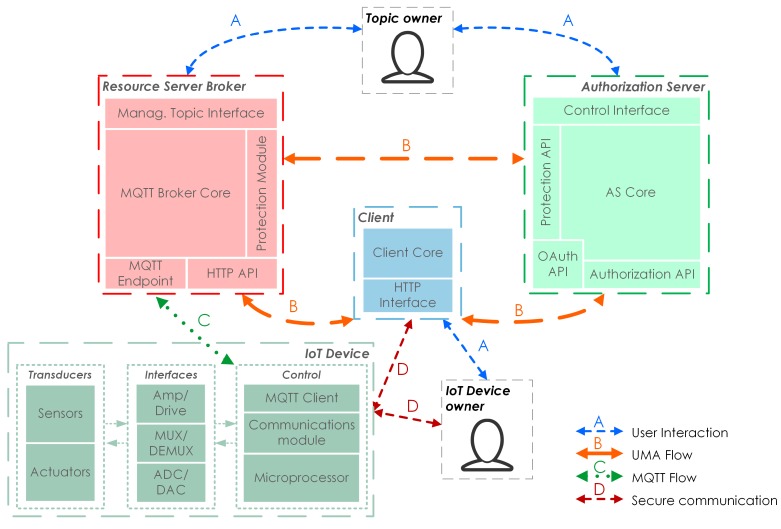
The main diagram of the proposed solution. ADC: Analog to Digital Converter; DAC: Digital to Analog Converter; DEMUX: Demultiplexer; HTTP: Hypertext Transfer Protocol; IoT: Internet of Things; MUX: Multiplexer.

The main components of the proposed solution are shown in Figure 3. There are four communication channels between the different elements:

- User interaction (A): Interaction between a user and a component through an interface.
- UMA flow (B): Set of requests and responses between the components defined in the UMA protocol.
- MQTT flow (C): Set of requests and responses between the MQTT client and broker defined in the protocol.
- Secure communication (D): Communication channel for the configuration of an IoT device.

In the following sections, we are going to describe the functional entities and flows involved in the proposal.

### 3.1. Functional Entities

- Resource: MQTT topics are modelled as UMA resources. A group of MQTT topics with the same authorization requirements can be seen as a Resource Set (RS). Given that MQTT topics form an RS, they will have a set of access policies associated. These policies must be defined by the topic owner in the AS, and will have to be evaluated to allow or deny the publishing and subscription actions, depending on who starts them.
- Resource Server Broker: This must include the MQTT Broker functionality and APIs, along with UMA-specific interfaces and APIs required by the UMA Resource Server flow.
- Authorization Server: In our proposal, this component is defined exactly as in the UMA specifications. Access control policies will be defined in this server and will need to be built considering the particularities of the resources proposed in this solution (MQTT topics).
- Client: An application that allows the IoT device owner to obtain a valid RPT through the UMA authorization flow. The IoT device owner must transmit the RPT to the device in a secure way. Typically, this application can be accessed via a Browser or using a Mobile App.
- IoT Device: Data generator or consumer that uses the MQTT protocol publish or subscribe actions to the Resource Server. All actions (to either publish or subscribe) must be accompanied by the RPT and will be defined by the IoT Device Owner.

### 3.2. Functional Flows

As described in the UMA specification [4], the access control process can be divided into three phases. In this section, we will describe how these flows are used in our proposal.

#### 3.2.1. Phase 1—Protecting a Topic

As we have explained before, MQTT topics are modelled as UMA resources. Right after a new topic is defined, it must be registered for protection. Under our proposal, the creator of a topic will also be the topic owner. From the UMA point of view, the topic creator will be the Resource Owner. A set of rules has to be observed when protecting a new topic:

1. In MQTT, topics follow a hierarchical tree structure. A protected topic will be defined as a complete route from a root to a leaf.
2. A previously protected topic cannot be protected again.
3. A child topic cannot be protected if the user has not permissions over the parent topic.
4. The protection applied to a topic is applied to it and all its descendants.

Once the owner has defined the new topic as a UMA resource, the remaining process to protect it is the same as defined by the UMA specification.

#### 3.2.2. Phase 2—Getting Authorization

After the topic has been registered, any subscription or publishing action will follow the authorization flow, as defined by UMA. This phase must be started by a user (in our use case, the IoT Device Owner). After this phase, the IoT Device Owner acquires an RPT Token, with the necessary permission associated with it.

If the IoT Device Owner needs to add or update permissions associated to the RPT, this flow can be used again without performing all the steps; the AAT and RPT tokens are already stored in the client while the session between the IoT Device Owner and the client lasts.

#### 3.2.3. Phase 3—Performing MQTT Actions

In this phase, the previously acquired RPT Token is used to access the resource, to publish and/or subscribe using the topic. The IoT Device Owner must configure his/her devices including the valid RPT obtained in phase 2 and must define which actions to perform. From this moment on, devices can publish or receive data to the configured topic without any further IoT Device Owner intervention.

**Figure 4:**
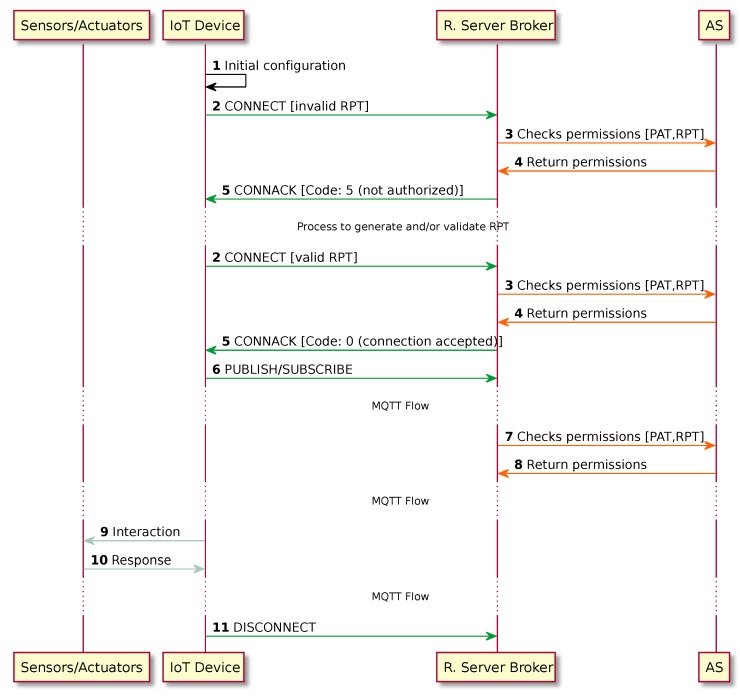
Sequence diagram for accessing the protected Message Queue Telemetry Transport (MQTT) flow.

It is important to notice that phase 3 of the UMA specification “is effectively the success path embedded within phase 2” [4]. Full message flow is shown in Figure 4 and is described below.

1. Initial Configuration: The IoT device must contain the information defined by the IoT Device Owner such as the Resource Server Broker location, topic name, actions to perform, etc. This information can be stored during the IoT device manufacturing process and can be modified later by the IoT Device Owner. In our proposal, it is mandatory that all devices contain:
  (a) MRF_id: Unique identifier of the device manufacturer.
  (b) Device_id: Unique identifier of the device.
  (c) SN: Unique Serial Number composed by a 8-bit Unicode Transformation Format (UTF-8) encoded alphanumeric string.
2. CONNECT: The IoT device, following the MQTT protocol, starts the flow, defined by the actions configured by its owner. The IoT device will send a CONNECT message to the Resource Server Broker. This message must include the RPT.
3. Check Permissions: Using the RPT in the request and the Protection API Token (PAT) associated to the service, the Resource Server Broker performs an OAuth Introspection [46] at request, including the list of scopes associated to the RPT.
4. Return permissions: The AS evaluates the policies. To do this, it has to take into account the RS identity (identified by the PAT), the requester identity (identified by the RPT), and the specific list of scopes included in the request. The AS will then create a list of permissions that will be returned to the Resource Server Broker.
5. CONNACK: The Resource Server Broker binds these permissions to the open session between the IoT device and itself. It will return a CONNACK message. If the RPT issued in the request has not been validated, the AS response (step 4) will be an empty list. In that case, the Resource Server Broker will answer with a code 5 in the header of the CONNACK message. This indicates that the IoT device that is not authorized and that it must start the RPT validation process. This process is explained in Section 3.2.4.
6. PUBLISH/SUBSCRIBE: The IoT device will perform the actions described by its configuration.
7. Check Permissions: As in step 3, the Resource Server Broker performs an OAuth Introspection request including the list of scopes associated to the publish/subscribe actions.
8. Return Permissions: As in step 4, the AS will then create a list of permissions after evaluating them.
9. Interaction: In this step, the IoT device can publish and subscribe to MQTT queues, and perform the required interaction with its sensors and actuators.
10. Response: The sensors and actuators will answer according to the actions performed.
11. DISCONNECT: Once all actions have been performed, the IoT device will close the MQTT session by sending a DISCONNECT message to the broker.

#### 3.2.4. RPT Validation Process

Due to the heterogeneity of IoT devices, the tokens used for message transmission can be generated and stored using different methods. The simplest scenario implies that the generation and storage of the token in the device is performed during its manufacturing process, but it might not always be the case. In this section, we propose a mechanism for RPT generation and validation which allows token management after the devices are deployed.

**Figure 5:**
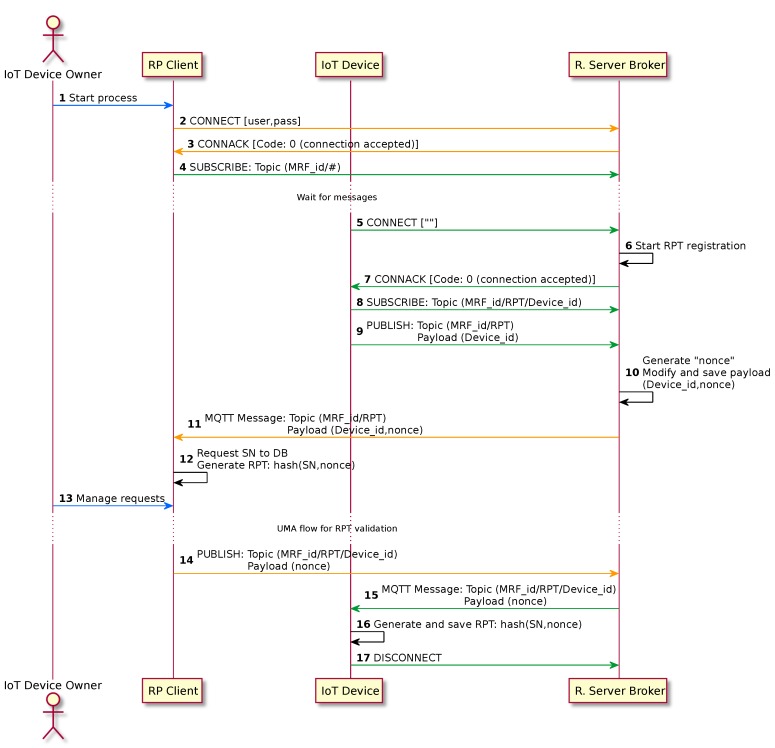
Sequence diagram for Requesting Party Token (RPT) validation. DB: Database; MRF: Manufacturer; SN: serial number.

The process will start with an IoT device which is not provided with a validated RPT, and will also be performed if the RPT is rejected when trying to stablish an authorized connection (see step 5 in Section 3.2.3). In Figure 5 we show the requests and responses used during this process, and they are further explained below.

1. Start Process: Following the methodology defined in OAuth 2.0, the IoT Device Owner is the user that starts the process by connecting himself to the client which will perform requests in its name.
2. CONNECT (from the RP Client): The client performs an MQTT connection with the Resource Server Broker using the IoT Device Owner credentials.
3. CONNACK (to the RP Client): The Resource Server Broker checks the user credentials from the client and, if they are correct, answers with a CONNACK message using the 0 code in its header.
4. SUBSCRIBE (from the RP Client): Once the client completes the connection with the Resource Server Broker, he/she subscribes him/herself to the specific topic of its manufacturer (“MRF_id/#”). The credentials used in the connection step will only allow this subscription, depending on the manufacturer identifier owned by the user.
5. CONNECT (from the IoT device): After the IoT device has performed the first connection, or receives a CONNACK (with Code 5, meaning not being authorized), the device starts a connection process using an empty string as an RPT.
6. Start RPT registration: When the Resource Server Broker receives a connection request with an empty RPT, it stores the client identifier and allows it to subscribe and publish in certain specific topics.
7. CONNACK (to the IoT device): The Resource Server Broker responds to the connection with a CONNACK and Code 0 to the request issued in step 6.
8. SUBSCRIBE (from the IoT device): The IoT device subscribes itself to the topic composed by the manufacturer identifier and its own identifier (“MRF_id/RPT/Device_id”), and waits for the RPT validation process to finish.
9. PUBLISH (IoT device): By publishing its own identifier in the “MRF_id/RPT” topic, the IoT device advertises that it wants to start the new RPT validation process.
10. Generate “nonce”: The authorization module of the Resource Server Broker, after receiving a message in the topic “MRF_id/RPT”, generates a random number to be used only once in each RPT generation (a “nonce”) and stores it temporally. This number is included in the payload of the MQTT message sent in the next step.
11. MQTT Message (to the RP Client): The request for the RPT validation process beginning is sent to the RP client through the previous subscription to the “MRF_id/#” topic (step 4).
12. Generate RPT (in the RP Client): Using the Device_id, the RP Client looks for the corresponding Serial Number (SN) in its database. Using the SN and the “nonce” received in the previous step, the client generates a RPT by applying a hash function. The specific function to use should be previously configured by the IoT Device Owner depending on the device restrictions and its own preferences.
13. Manage Requests: RPTs generated in step 12 are stored in the RP Client until they are confirmed by the IoT Device Owner. The RPT verification process is the one specified in UMA. The RPT validation can be performed both individually or in batch mode. Also, the IoT Device Owner can previously define in the AS a set of policies to be applied to the RPTs, which would ease the configuration in this step.
14. PUBLISH (from the RP Client): Once the RPT is provided with the defined permissions after its validation, the RP Client publishes each “nonce” in the corresponding topic (“MRF_id/RPT/Device_id”). This is the way the RP Client advertises the correct ending of the validation process.
15. MQTT Message (to the IoT device): The IoT device receives a message from the topic to which it was subscribed in step 8.
16. Generate RPT (in the IoT device): In this step, the IoT device already knows that the RPT candidate has been validated. Using the SN which is stored in it, and the “nonce” received in the previous step, the hash function is executed to obtain the RPT value. The hash function used must be the same used in step 12.
17. DISCONNECT: When all actions have been performed, the IoT device closes the MQTT session by sending a DISCONNECT message to the broker. After this process, the IoT device will perform another connection with the Resource Server Broker using the validated RPT.

### 3.3. Energy Consumption Considerations

As stated above, energy consumption is one of the main challenges to address in this type of scenario. Usually, IoT devices need to work autonomously or are located in places with difficult physical access. In these cases, the energy consumption is a critical factor. The survey in [47] shows that energy efficiency is related to network and application layer protocols used in IoT systems. In the evaluation of our proposal we will address this matter.

## 4. Experiments and Results

We have developed a prototype implementation of the proposal in order to validate it. This section is divided into three sections. The first and second describe the implementation and set-up of each element used in the proposal experiments and the deployment platform used for them. The tests related with the energy consumption will be described in Section 4.1.1.

### 4.1. Implementation and Set-Up

Nowadays, there are many commercial solutions which allow the deployment of IoT devices. For instance, Amazon Web Services IoT Core (AWS IoT Core) [48] and The Things Network [49] offer proprietary platforms which include mechanisms for communications, authentication, and authorization in IoT scenarios. These solutions propose the integration of all these services in a single platform, which limits the interoperability with other systems. Regarding the most used MQTT broker implementations, we can cite HiveMQ [50] and Mosquitto [51]. The first one offers native authentication, authorization, and permissions services, such as X.509 client certificate authentication, username and password authentication and authorization, client ID authentication and authorization, and IP-based authentication and authorization. On the other hand, Mosquitto integrates a username and password authentication scheme, and allows developers to implement third-party authorization plugins, which allow a high degree of customization.

This prototype implementation has focused on the input and output messages generated by an IoT device, giving that is the most constrained element in the system.

All the entities defined in Section 3.1 have been developed separately and deployed using Web Servers (cloud AWS servers), Arduino devices, and NodeMCU v1.0 boards [52]. This prototype intends to represent an IoT environment.

The implementation of each entity is described below:

- Resource Server Broker: Is composed of two internally connected servers. The MQTT functionalities are provided by a deployed Mosquitto broker. Mosquitto allows the creation and utilization of plug-ins for authorization customization. We have chosen the mosquitto_pyauth plug-in which allows the creation of Python scripts to modify the Mosquitto authorization scheme. The HTTP API used to connect the Resource Server with the client, the Protection module used to connect the Resource Server with the Authorization Server, and the topic management interface have been developed in a single server.
- Authorization Server: Is a Web Server written in Python. The implementation includes UMA APIs, a user interface for control and policy definition, and an OAuth API.
- Client: Is a web application that provides a user interface for the configuration of IoT devices and allows communication with the Resource Server HTTP API and the Authorization Server’s Authorization API.
- IoT devices: Are deployed in the NodeMCU v1.0 board using a MQTT library for Arduino. This development board is based on Wi-Fi MCU ESP8266.
- Measuring Device: Is composed of an Adafruit INA219 [53] High Side DC Current Sensor Breakout board to measure the energy consumption and an Arduino Uno R3 board with a Arduino Ethernet Shield to collect data.

The entities described in Section 4.1 have been deployed in the following way:

- Resource Server Broker and Authorization Server: Both are deployed in a cloud environment. Specifically, we have chosen AWS as the cloud provider. We created two EC2 t2.micro (1 vCPUs, 2.5 GHz, Intel Xeon Family, 1 GiB memory) machines deployed in the EU-West 1 area and configured in the same subnet. The machines run Ubuntu Server 14.04 as the operative system.
- Client: Has been deployed in a local server with Internet access.
- IoT Device: Has an active Wi-Fi interface and has Internet access.
- Measuring Device: Collects and stores the energy consumption measurements and return this information in a local server.

#### 4.1.1. Energy Consumption Measurement Configuration

We have divided the energy consumption tests according to their objectives: The first set of tests tries to validate the election of a message-based communications system versus other alternative communications systems, and the second set evaluates the energy consumption overhead when adding the access control system to the MQTT protocol.

In the following paragraphs we will show the specific definition of each set of tests:

- **Validation of the communications system**:
  1. Selection of two well-known Internet-based communication systems: REST API vs. message-based communications (MQTT).
  2. Configuration of an IoT device as a Web Server to use a REST API and another device as an MQTT client, both with the exact same characteristics.
  3. Definition of a common set of message exchange that allows the measurement of the energy consumption in each device.
  4. Measurements start when the first message is exchanged and stop when all the messages configured have been successfully delivered.
- **Energy consumption overhead measurements**:
  1. Configuration of two IoT devices as MQTT clients. Both devices have the exact same characteristics.
  2. Configuration of one Broker as a standard MQTT Mosquitto Broker (without authorization) and other as a Resource Server Broker using the access control proposed in this paper.
  3. Definition of a common set of message exchange that allows the measurement of the energy consumption in each device.
  4. Measurements start when the first message is exchanged and stop when all the messages configured have been successfully delivered.

### 4.2. Implementation Results

In this section, we present the results obtained after testing our prototype implementation, following the goals defined in the Section 4.1. A test benchmark has been established to assess the response times in the proposed scenario. This benchmark starts from an initial set up with the following characteristics:

- All the involved devices are powered up and in a stable state.
- The IoT device MQTT client has a valid RPT (used for the action of subscribing and publishing).
- The IoT device MQTT client has performed a subscribing action to a specific test queue.
- The RS Broker has validated the subscription by verifying its permissions.

The test consists in performing a complete cycle of publication and reception (with a previous subscription) of an MQTT message. The IoT device publishes a message to the test queue and waits for its reception. To perform measurements, two locations have been selected:

- In the IoT device the measurement starts when it performs the publishing action, and stops when it confirms the message reception.
- In the RS Broker the measurement is performed inside of the PyAuth authorization module. It starts when the permission validation request is sent to the AS (OAuth Introspection flow) and stops once the response is received.

To obtain a reference measurement for each scenario, the same cycle has been performed in each one, disabling the authorization module. This way, it is possible to determine the processing time for each MQTT message by the Mosquitto Broker.

The following equations express the time measurements obtained from the IoT device:

(1) $$T_{T\_NoAuth}=T_{P\_IoTD}+T_{Tx\_Net}+T_{P\_RSB}$$

(2) $$\begin{array}{c} T_{T\_Auth}=T_{P\_IoTD}+T_{Tx\_Net}+T_{P\_RSB}+T_{P\_PyAuth}+2\times T_{P\_Intros} \end{array}$$

Equation (1) represents the complete test cycle time when the authorization module is not active. It consists of the sum of the IoT device processing time ($T_{P\_IoTD}$), the packet transmission through the specific network ($T_{Tx\_Net}$) and the Mosquitto RS Broker processing time ($T_{P\_RSB}$). The same cycle time measurement, but when the authorization module is active, is represented by Equation (2), where the partial time of the PyAuth authorization module ($T_{P\_PyAuth}$) and the time needed to obtain the AS validation response ($T_{P\_Intros}$), which includes both the processing time of the validation in the AS and packet transmission time, are incorporated. There are two introspection requests in each cycle: one for the publishing action and another for the subscribing actions.

Applying Equations (1) and (2), it is possible to obtain the additional time imposed by the authorization module ($T_{P\_PyAuth}$) as shown in Equations (3) and (4).

(3) $$T_{T\_Auth}=T_{T\_NoAuth}+T_{P\_PyAuth}+2\times T_{P\_Intros}$$

(4) $$T_{P\_PyAuth}=T_{T\_Auth}-\left(T_{T\_NoAuth}+2\times T_{P\_Intros}\right)$$

This same cycle has been repeated 1000 times to obtain a representative mean value of each measurement. These results, accompanied with standard deviation values, are shown in Table 2.

#### 4.2.1. Energy Consumption Results

In this section we have performed the tests defined in Section 4.1.1. During the experiments, the development board has been monitored (voltage and current values) using the INA219 sensor. In Table 3 we show the mean values for current measured by the sensor when performing each experiment. In Table 4 the consumption results for each configuration are shown.

Table 3 shows the mean value and the standard deviation for electric current consumption from the development board NodeMCUv1 when we simulate the transmission and reception of 1000 messages using the following configurations:

- Idle state (Wi-Fi off): Development board with disabled Wi-Fi interface, in idle state during the average time it takes to send and receive 1000 messages in the other experiments.
- Idle state: Development board with enabled Wi-Fi interface, but still in an idle state during the average time it takes to send and receive 1000 messages in the other experiments.
- HTTP Client (with requests): Wi-Fi interface enabled and performing the transmission and reception task for 1000 messages with the HTTP following the REST architecture.
- MQTT Client (with requests): Wi-Fi interface enabled and performing the transmission and reception task for 1000 MQTT messages using the default protocol configuration, and disabling the authorization module both in the IoT device and the Broker.
- MQTT Client (with requests and authorization module): Wi-Fi interface enabled and performing the transmission and reception task for 1000 MQTT messages using the proposed configuration. That is, enabling the authorization module in both the IoT device and the Broker.

Besides the current values shown in Table 3, we have carried out the same mean calculation using the voltage used to feed the development board. The mean value obtained is 5.08 V with a standard deviation of 0.07.

Using the resultant values, we can obtain the energy consumption for each message transmission in each configuration by applying Equation (5), where $V_{cc}$ is the voltage (in volts), $I_{c}$ is the current (in amps) measured during the message transmission, and $t_{m}$ is the time needed (in seconds) for the message to be fully transmitted.

(5) $$E_{c}=V_{cc}\times I_{c}\times t_{m}\left[J\right]$$

In Table 4 we show the energy consumption (in millijoules) needed for the transmission of a single message in each one of the configurations used in the experiments where there is actual message transmission.

## 5. Discussion

In this section we will analyse the results shown in Section 4.2 and then we will extract the conclusions derived from them and about our proposal.

Data from Table 2 show the possible delays introduced by the proposal once the access control layer is added to the MQTT protocol. Following these results, we can conclude that the processing time of the authorization module represents a very small part of the total communication time (only 0.23 ms).

To keep these processing times close to this value, it is possible to apply the solutions which are being used in Internet-based access control schemes. In those scenarios, it has been proven that the federated access control systems allow the replication of authorization servers, which limit the workload of each one of them, while maintaining response times between acceptable values.

The proposed solution adds the validation of the access control permissions in each action. This time is measured in $T_{P\_Intros}$. In a cloud environment such as the one deployed for these tests, the obtained values are only around the 15% of the total.

This value can be limited in cloud environments by using the correct configurations to reduce the latency between the services that compose the system (placing the services in cluster of the same geographic zone, enabling priority routes, etc.).

Additionally, using the obtained results, shown in Table 4, we can conclude the following:

- **Validation of the communications system**: With the same number of messages and the same conditions, using a message-based communication system, energy consumption is lower than for a REST-based system. This is due to the simplicity of the MQTT protocol and the lower amount of redundant information sent when compared with the HTTP.
- **Energy consumption overhead measurements**: From the MQTT client perspective, the actions performed to communicate with and without an access control system are the same (only needing to add the credentials to the headers of the message). As shown in lines 4 and 5 of Table 3, the current measurements for MQTT with and without authorization are very close. From these values and the results shown in Table 2, it is possible to obtain the difference in consumption between sending messages with and without authorization (as shown in Table 4). This consumption overhead is bound to the extra time needed for the Introspection request ($T_{P\_Intros}$). This consumption can be reduced if the device is put into an idle state when it is not preforming any activity (for instance, between sending and receiving messages, when there is no sensor monitoring, etc.).
  This is shown in the second and third line of Table 4, where the average of the values is almost the same.

The goal of this paper is to present a unified authorization system for an IoT environment. To achieve this, we have proposed modelling the key elements that compose a message exchange protocol such as MQTT (i.e., the message queues, called topics) as resources. An authorization system based on a OAuth 2.0 profile such as UMA can be used to protect these “resources” as it protects Internet-based services. Analysing the results obtained in Section 4.2, and combining them with the delay introduced by the proposal and energy consumption measurements shown in Table 2 and Table 4, we can assure the viability of the proposed solution. We can also conclude that in a conventional IoT environment using a cloud-based deployment, the overhead produced by the authorization schema is acceptable when compared with the advantages of using this approach.

Regarding the management of the access control associated with the IoT devices, our proposed solution enables the management of permissions without interacting physically with the device. The IoT Device Owner only needs to know the RPT of a specific device to change the access policies in a cloud server.

It is important to highlight that once a Broker is protected with our solution, all messages exchanged through it are protected by default. This means that any registered topic has the permissions defined by its owner using access control policies. A topic without defined access control policies cannot be accessed. This guarantees a high protection level against insecure default configurations.

Finally, we can conclude that our proposal improves other existent solutions as it is based on the protection of communication procedures without being bound to a specific platform or system. Other proposals perform data protection by following platform-specific access control schemes (AWS IoT Core, The Things Network). There are also MQTT brokers such as HiveMQ which offer mechanisms for filtering the publishing or subscription to certain topics using authentication and authorization mechanisms like X.509 client certificates or user credentials. These techniques require specific permission configurations, which might make them more difficult to apply in big complex deployments of IoT devices, and they cannot be reused in different scenarios. Our proposal shows the advantages of abstracting the actions, methods and characteristics of communication procedures as resources during the whole life cycle of the access control system. We apply this method to MQTT in this paper, but it can be extended to any other communications protocol. Also, this method assures the correctness of the information flow as it is based on the OAuth 2.0 access control scheme, and eases the management of IoT devices permissions through the definition of policies in an external Authorization Server, which could be previously defined for Web-based services.

As future work, we consider three main research lines. The first one would be to generalize the proposed solution to other IoT protocols (the CoAP, AMQP, REST, etc.). This will allow us to analyse the viability of applying the abstraction as a resource for key elements following the proposal of this paper. The second line would be to explore possible encryption systems that allow for MQTT communications to be secure in insecure environments. Finally, the last line would be to perform a specific study of the application of access policies used in Internet-based services to protect the message exchange between IoT devices.

## Figures and Tables

**Table 1:** Tokens used in User-Managed Access (UMA). API: Application Programming Interface; AS: Authorization Server; RP: Requesting Party.

Token	Used by	To Access	Goal/s
Authorization API Token (AAT)	RP Client	AS Authorization API	Request an RPT
Protection API Token (PAT)	Resource Server	AS Protection API	Register resources or check permissions
Requesting Party Token (RPT)	RP Client	A resource in a Resource Server	Access a UMA-protected resource

**Table 2:** Results in delay measurement (milliseconds). $T_{T\_NoAuth}$: Time without using the authorization module; $T_{T\_Auth}$: Time using the authorization module; $T_{P\_Intros}$: Time needed to obtain the AS validation response; $T_{P\_PyAuth}$: Time imposed by the authorization module.

Value	Mean	Standard Deviation
$T_{T\_NoAuth}$	43.83	5.99
$T_{T\_Auth}$	67.74	11.07
$T_{P\_Intros}$	11.84	1.28
$T_{P\_PyAuth}$	0.23	-

**Table 3:** Current measures during the experiments (milliamperes).

Developer Board	Wi-Fi	Mean	Standard Deviation
Idle state	Off	33.47	1.67
Idle state	On	81.08	4.21
HTTP Client (with requests)	On	96.37	7.38
MQTT Client (with requests)	On	88.48	2.40
MQTT Client (with requests and auth.)	On	88.51	2.27

**Table 4:** Results in energy consumption measurement (millijoules). REST: Representational State Transfer.

Experiment	Mean	Standard Deviation
REST request	33.16	5.71
MQTT request	19.70	1.01
MQTT request with auth.	30.46	1.75

## Abbreviations

The following abbreviations are used in this manuscript:

6LoWPAN	IPv6 over Low power Wireless Personal Area Networks
AAT	Authorization API Token
ABAC	Attribute-Based Access Control
ACLs	Access Control Lists
ACM	Access Control Matrix
AMQP	Advanced Message Queuing Protocol
API	Application Programming Interface
AS	Authorization Server
AWS	Amazon Web Services
CapBAC	Capability-Based Access Control
CoAP	Constrained Application Protocol
CoRE	Constrained RESTful Environments
DDS	Data Distribution Service
DNS-SD	Domain Name System Service Discovery
HTTP	Hypertext Transfer Protocol
IEEE	Institute of Electrical and Electronics Engineers
IETF	The Internet Engineering Task Force
IoT	Internet of Things
IPv6	Internet Protocol version 6
M2M	Machine to Machine
mDNS	multicast Domain Name System
MQTT	Message Queuing Telemetry Transport
OAuth	Open Authorization
OrBAC	Organizational-Based Access Control
PAT	Protection API Token
QoS	Quality of Service
RBAC	Role-Based Access Control
REST	REpresentational State Transfer
RO	Resource Owner
RP	Requesting Party
RPL	IPv6 Routing Protocol for Low-Power and Lossy Networks
RPT	Requesting Party Token
RS	Resource Set
SSL	Secure Sockets Layer
TLS	Transportation Layer Security
UCON	Usage Control
UMA	User-Managed Access
UTF-8	8-bit Unicode Transformation Format
VPN	Virtual Private Network
XACML	Extensible Access Control Markup Language
XML	eXtensible Markup Language
XMPP	Extensible Messaging and Presence Protocol
